# Synchronous breast carcinoma with ipsilateral axillary tuberculosis, posing a diagnostic dilemma

**DOI:** 10.1093/jscr/rjaf218

**Published:** 2025-04-19

**Authors:** Felix A Raj, Imran T Ajmal, Amrithraj Thiyagarajan, M Y Ajmal, Pavithra Selvam, Venkiteswaran Muralidhar

**Affiliations:** Department of General Surgery, Chettinad Hospital and Research Institute, Chettinad Academy of Research and Education, Rajiv Gandhi Salai, Kanchipuram, Tamil Nadu 603103, India; Department of General Surgery, Chettinad Hospital and Research Institute, Chettinad Academy of Research and Education, Rajiv Gandhi Salai, Kanchipuram, Tamil Nadu 603103, India; Department of General Surgery, Chettinad Hospital and Research Institute, Chettinad Academy of Research and Education, Rajiv Gandhi Salai, Kanchipuram, Tamil Nadu 603103, India; Department of General Surgery, Chettinad Hospital and Research Institute, Chettinad Academy of Research and Education, Rajiv Gandhi Salai, Kanchipuram, Tamil Nadu 603103, India; Department of General Surgery, Chettinad Hospital and Research Institute, Chettinad Academy of Research and Education, Rajiv Gandhi Salai, Kanchipuram, Tamil Nadu 603103, India; University of Birmingham, Department of Bioinformatics, Birmingham, United Kingdom

**Keywords:** extrapulmonary tuberculosis, invasive breast carcinoma, axillary node, anti-tubercular therapy

## Abstract

This case report describes a 63-year-old postmenopausal diabetic female with synchronous primary invasive ductal carcinoma of the right breast and tuberculosis (TB) of the ipsilateral axillary lymph nodes. Presenting with a palpable right breast lump and axillary lymphadenopathy, the patient underwent imaging, revealing BIRADS V classification and suspicious lymph nodes. Core needle biopsy confirmed invasive carcinoma, while lymph node histology revealed caseating granulomatous inflammation consistent with TB. Managed with modified radical mastectomy, adjuvant chemotherapy, and antitubercular therapy, the patient showed complete disease resolution on follow-up. This rare coexistence underscores the necessity for histopathological confirmation and multidisciplinary management to optimize outcomes.

## Introduction

The coexistence of breast carcinoma and tuberculosis (TB) in the axillary lymph nodes is an exceedingly rare clinical presentation, often posing significant diagnostic and therapeutic challenges. Breast carcinoma is the most common malignancy affecting women worldwide, while tuberculosis remains a significant public health concern, particularly in developing nations [1]. The synchronous occurrence of these two conditions in the same anatomical region is unusual, as they typically have distinct pathophysiological mechanisms and risk factors [2]. This case report highlights the importance of meticulous clinical evaluation and the need for histopathological confirmation to differentiate between metastatic malignancy and granulomatous inflammation in axillary lymph nodes. Misdiagnosis or delay in diagnosis can lead to inappropriate treatment, impacting prognosis [3].

Breast cancer is a leading cause of cancer-related morbidity and mortality among women globally [4]. On the other hand, tuberculosis, primarily a pulmonary infection, can involve extra-pulmonary sites such as lymph nodes, especially in endemic regions. Axillary lymphadenopathy in a patient with breast cancer is often presumed to be metastatic until proven otherwise. However, granulomatous diseases like tuberculosis should also be considered in the differential diagnosis, particularly in areas where TB is endemic [5].

The literature reports only a few cases of synchronous breast carcinoma and axillary TB, emphasizing the rarity of this clinical entity [1, 4]. A comprehensive histopathological and microbiological examination of lymph nodes is crucial to establish the diagnosis and guide appropriate treatment. Failure to recognize the coexistence of these conditions can lead to suboptimal outcomes, as the management strategies for carcinoma and tuberculosis differ significantly [3, 5].

## Case report

The patient, a 63-year-old woman, presented with a lump in her right breast persisting for 20 days. The lump was not associated with pain, nipple discharge, or inversion. Clinical examination revealed a palpable mass in the upper outer quadrant of the right breast along with palpable, firm axillary lymph nodes. No other systemic complaints were noted. She had a medical history of diabetes mellitus and hypertension managed with medications. Her surgical history included bilateral cataract surgery. There was no personal or family history of malignancy, tuberculosis, or other significant systemic diseases.

**Figure 1:**
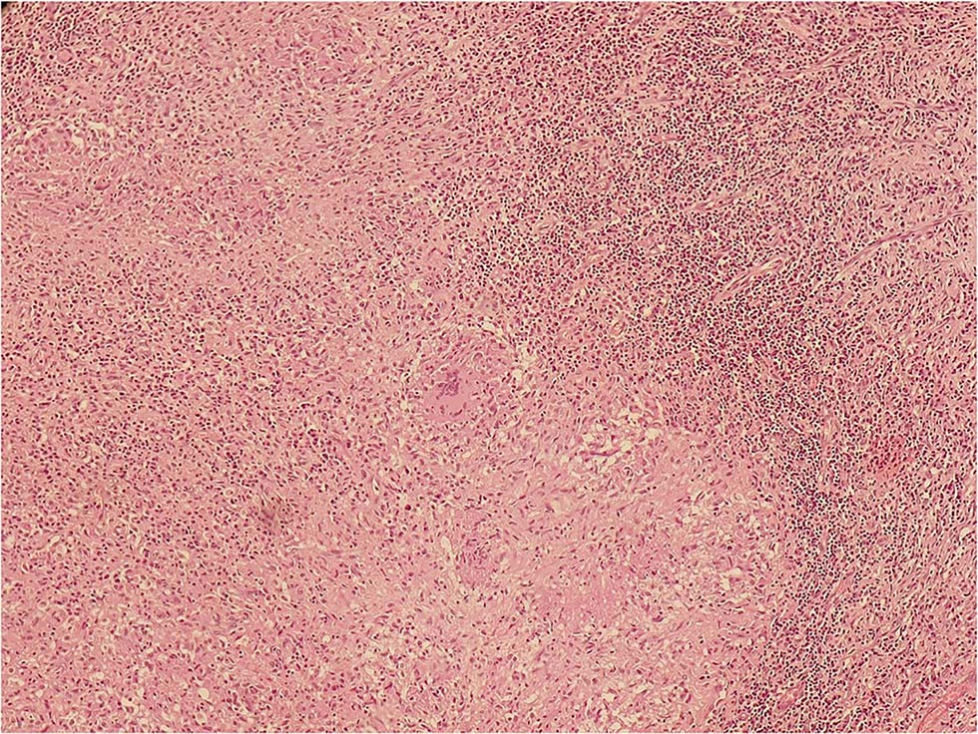
Histopathological slide section of biopsy specimen taken from the right axillary node lesion showing caseating granulomatous inflammation, consistent with tuberculosis.

**Figure 2:**
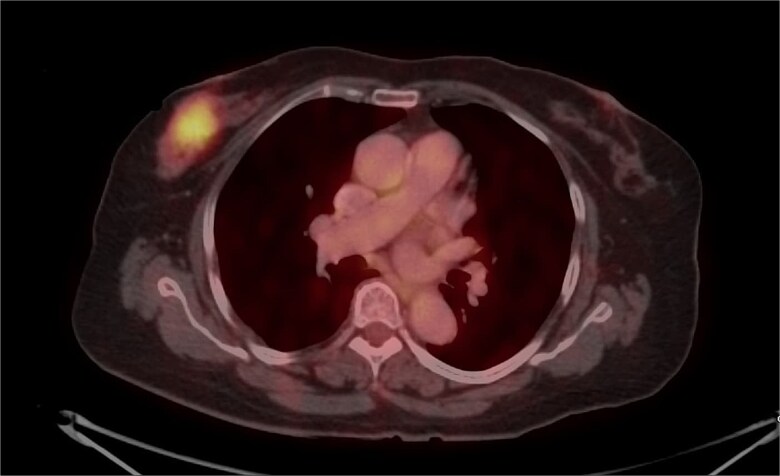
PET-CT axial view of the thorax, indicating a metabolically active lesion in the right breast.

**Figure 3:**
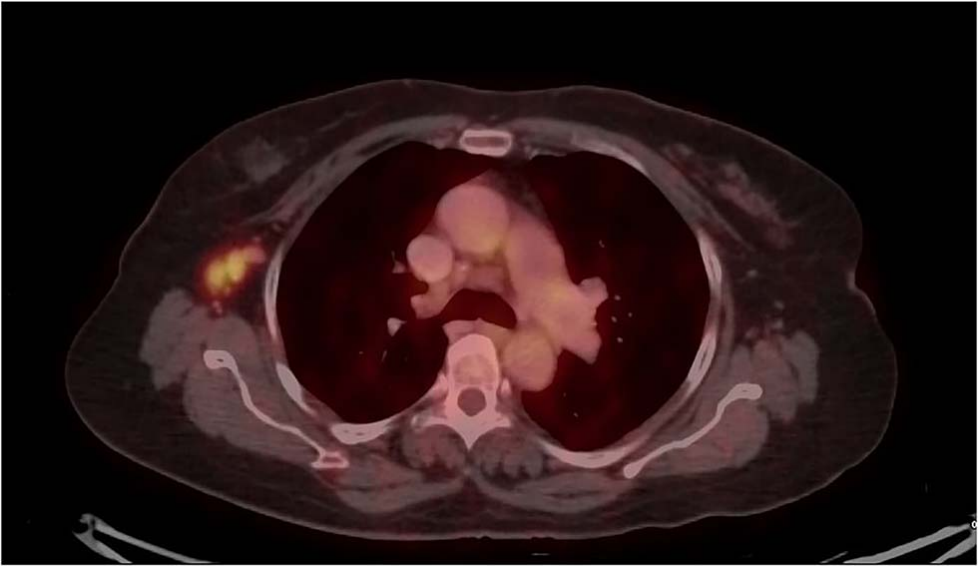
PET-CT axial view of the thorax, indicating a metabolically active lesion in the right axillary region, involving the right axillary lymph nodes.

Mammography revealed an asymmetrical density in the upper outer quadrant of the right breast with BIRADS V classification. Ultrasonography identified an irregular hypoechoic lesion measuring 3.8 × 2.5 cm in the right breast. Additionally, multiple enlarged axillary lymph nodes with cortical thickening were seen, the largest measuring 1.02 cm in diameter. A core needle biopsy of the breast lesion demonstrated features of invasive carcinoma, not otherwise specified (NOS), Grade II based on the Nottingham grading system. Immunohistochemistry revealed the tumor to be ER-positive (78%), PR-negative, Her2Neu-negative, and Ki67–21%, confirming it as Luminal A subtype. The excised axillary lymph nodes exhibited caseating granulomatous inflammation, consistent with tuberculosis (Fig. 1). Acid-fast bacilli (AFB) testing further confirmed the diagnosis of TB. Pre-surgical imaging revealed metabolically active areas in the breast lesion and axillary lymph nodes with no evidence of systemic metastases (Figs 2 and 3).

**Figure 4:**
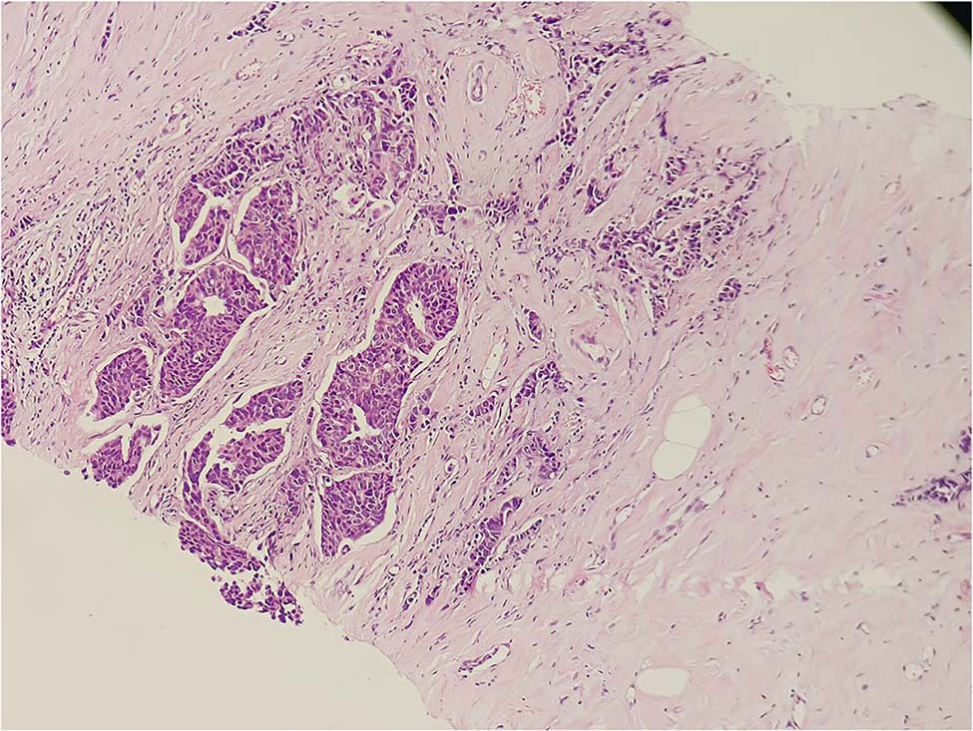
Histopathological slide section of biopsy specimen taken from the right breast lesion showing invasive ductal breast carcinoma, NOS, grade II based on the Nottingham grading system.

The patient was diagnosed with invasive carcinoma of the right breast (pT2N0, Stage IIA) and ipsilateral axillary lymph node tuberculosis. The patient underwent a right modified radical mastectomy (MRM) with axillary dissection. Histopathological evaluation of the breast specimen confirmed invasive carcinoma (Fig. 4). The resected margins and nipple-areola complex were free of tumor infiltration. Adjuvant chemotherapy was administered in six cycles (anthracycline-based regimen), followed by hormonal therapy with letrozole due to ER positivity. The patient received standard anti-tuberculosis therapy (ATT) for 6 months as per national guidelines. Subsequent PET-CT scans demonstrated a reduction in soft tissue thickening and no evidence of metabolically active disease, signifying a good therapeutic response. The axillary tuberculosis also resolved with ATT.

## Discussion

The synchronous occurrence of breast carcinoma and axillary TB is a rare clinical entity that presents unique diagnostic and therapeutic challenges. This rare coexistence highlights the importance of a meticulous evaluation of axillary lymphadenopathy in patients with breast cancer, particularly in regions where TB is endemic. Breast carcinoma is the most common cancer in women globally, with lymphatic spread being a hallmark of disease progression [1]. On the other hand, TB, primarily a pulmonary infection, can involve extra-pulmonary sites, with lymphadenitis being the most common form of extra-pulmonary TB [3]. Axillary lymphadenopathy in patients with breast cancer is commonly attributed to metastatic disease, often overshadowing the possibility of alternative causes like TB.

Diagnosing synchronous breast carcinoma and TB requires a high degree of suspicion. Radiological imaging, while valuable in identifying lymph node abnormalities, is often insufficient in differentiating metastatic nodes from those affected by granulomatous inflammation [4]. Fine-needle aspiration cytology (FNAC) or excision biopsy with histopathological examination remains the gold standard for differentiating these pathologies [2]. In the present case, the axillary nodes were initially presumed metastatic based on imaging but were subsequently identified as tuberculous upon histological evaluation. The overlap of clinical symptoms and radiological findings between the two conditions can lead to diagnostic pitfalls. Features such as caseating granulomas in histology and the presence of acid-fast bacilli confirm TB, while cytological atypia and immunohistochemistry (IHC) findings support a diagnosis of breast carcinoma [5].

The coexistence of TB and carcinoma raises interesting questions about their pathophysiology. Some researchers have hypothesized that the immunosuppressive state induced by cancer or chemotherapy may predispose patients to opportunistic infections like TB [6]. Conversely, chronic inflammation caused by TB may create a microenvironment conducive to malignancy by inducing genetic instability or promoting angiogenesis [7].

The management of synchronous breast carcinoma and TB requires a multidisciplinary approach. The priority is to treat the malignancy effectively while addressing the infection to prevent complications. Surgery, adjuvant chemotherapy, and hormonal therapy constitute the backbone of breast cancer management, while a 6-month course of ATT remains the standard treatment for TB [8]. In the reported case, the patient underwent modified radical mastectomy and chemotherapy for the carcinoma, along with ATT for TB. Regular follow-ups with PET-CT imaging ensured the resolution of both conditions. The prognosis in such cases depends on early detection and appropriate management of both conditions. The presence of TB in lymph nodes should not deter oncological treatment, as TB typically responds well to ATT, even in immunocompromised states [9]. However, delays in diagnosing or managing either condition can adversely affect outcomes.

This case highlights the importance of considering TB as a differential diagnosis for axillary lymphadenopathy in breast cancer patients, especially in endemic regions. Histopathological confirmation is critical to avoid misdiagnosis and ensure appropriate management. The synchronous occurrence of these diseases, although rare, highlights the complex interplay between infection and malignancy.

## Conclusion

The synchronous occurrence of breast carcinoma and axillary TB, as demonstrated in this case, is an exceptionally rare entity that underscores the importance of thorough diagnostic evaluation. In this 63-year-old patient, the coexistence of invasive ductal carcinoma and caseating granulomatous inflammation in axillary lymph nodes presented unique diagnostic and therapeutic challenges. Meticulous histopathological and microbiological confirmation played a pivotal role in differentiating between metastatic carcinoma and granulomatous TB, enabling appropriate and timely treatment. The case emphasizes the necessity of a multidisciplinary approach, combining oncological treatment—including surgery, chemotherapy, and hormonal therapy—with antitubercular therapy to optimize outcomes. Notably, the simultaneous resolution of both conditions highlights the feasibility of concurrent management, even in complex scenarios. Regular follow-up with imaging ensured early detection of disease resolution and prevention of recurrence.
